# Natural Products for the Prevention and Treatment of Common Cold and Viral Respiratory Infections

**DOI:** 10.3390/ph16050662

**Published:** 2023-04-28

**Authors:** Nour Mammari, Quentin Albert, Marc Devocelle, Maša Kenda, Nina Kočevar Glavač, Marija Sollner Dolenc, Laura Mercolini, Jaroslav Tóth, Nagy Milan, Szilvia Czigle, Mihayl Varbanov, on behalf of the OEMONOM

**Affiliations:** 1 CNRS, L2CM, Université de Lorraine, 54000 Nancy, France; 2 INRAE, Aix Marseille Université, UMR1163 Biodiversité et Biotechnologies Fongiques, 13288 Marseille, France; 3 INRAE, Aix Marseille Université, CIRM-CF, 13288 Marseille, France; 4 SSPC (Synthesis & Solid State Pharmaceutical Centre), V94 T9PX Limerick, Ireland; 5 Department of Chemistry, Royal College of Surgeons in Ireland, RCSI University of Medicine and Health Sciences, 123 St. Stephen’s Green, D02 YN77 Dublin, Ireland; 6 Faculty of Pharmacy, University of Ljubljana, Aškerčeva Cesta 7, 1000 Ljubljana, Slovenia; 7 Research Group of Pharmaco-Toxicological Analysis (PTA Lab), Department of Pharmacy and Biotechnology (FaBiT), Alma Mater Studiorum—University of Bologna, Via Belmeloro 6, 40126 Bologna, Italy; 8 Department of Pharmacognosy and Botany, Faculty of Pharmacy, Comenius University Bratislava, Odbojárov 10, 832 32 Bratislava, Slovakia; 9 Laboratoire de Virologie, CHRU de Nancy Brabois, 54500 Vandœuvre-lès-Nancy, France

**Keywords:** herbal medicines, medicinal plants, common colds, therapy

## Abstract

The common cold is generally considered a usually harmless infectious disease of the upper respiratory pathway, with mostly mild symptoms. However, it should not be overlooked, as a severe cold can lead to serious complications, resulting in hospitalization or death in vulnerable patients. The treatment of the common cold remains purely symptomatic. Analgesics as well as oral antihistamines or decongestants may be advised to relieve fever, and local treatments can clear the airways and relieve nasal congestion, rhinorrhea, or sneezing. Certain medicinal plant specialties can be used as therapy or as complementary self-treatment. Recent scientific advances discussed in more detail in this review have demonstrated the plant’s efficiency in the treatment of the common cold. This review presents an overview of plants used worldwide in the treatment of cold diseases.

## 1. Introduction

The common cold is a conventional term for a mild upper respiratory illness (nose, sinuses, pharynx, and larynx), the catarrhal disorder, which may be viral or a mixed infection. The severity and type of symptoms vary depending on the immune status of the patient and the pathogen causing the infection. It generally involves a set of less-specific symptoms that include nasal stuffiness and discharge, sneezing, sore throat, cough, headache, malaise, and low-grade fever. The mucosal inflammation may involve the nasal mucosa, throat, sinuses, and larynx [1]. There is not a single cause for the illness; rather, it is a heterogeneous group of diseases caused by numerous viruses that belong to several different families. Occasionally, the common cold may spread to the lower respiratory tract, as well as predispose to bacterial complications [2,3,4,5,6].

The list of pathogens that may cause common cold symptoms includes rhinoviruses, respiratory syncytial virus, influenza viruses, parainfluenza viruses, coronaviruses, and adenoviruses. Less frequently, coxsackieviruses, echoviruses, bocavirus, Epstein-Barr virus, and human metapneumovirus are implicated [3,7]. Altogether, the condition is associated with more than 200 virus subtypes [8]. The common colds are distinctly different entities than rhinitis or pharyngitis, as well as influenza (flu). Colds are very common, especially in children [9]. The prevalence of common cold or viral acute rhinosinusitis (VAR) in the general population varies depending on different studies. Although VAR is a common problem, the precise incidence is difficult to estimate, and is often concluded as very high. It has been estimated that adults suffer two to five episodes of viral VAR (or colds) per year and school children may suffer seven to ten colds per year. Post-viral and bacterial VAR is less common. Approximately 0.5–2% of viral upper respiratory tract infections are complicated by bacterial infection [10,11].

Drug treatment is usually not necessary for colds. Some medications can, at best, help relieve the symptoms a little. Since colds are caused by viruses, antibiotic treatment is not warranted [12]. Antibiotics only fight bacteria [2]. Recommended therapy (allopathy) is mainly symptomatic: a combination of paracetamol, non-steroidal anti-inflammatory drugs—NSAIDs (reduce fever and soreness of the throat, and might also have some beneficial effect on cough), second-generation antihistamines with short-term benefit in reducing symptoms the first 2 days (reduce sneezing and rhinorrhea), nasal decongestants with small effect in nasal congestion in adults (reduce nasal blockage), ipratropium bromide (reduce rhinorrhea), probiotics, zinc when administered the first 24 h after the onset of symptoms, nasal saline irrigations, vitamin C (in selected patients with a suspected deficit or with high levels of physical activity), and some herbal medicines (Table 1) [3,10,11]. Frequent changes in the serotypes of the virus lead to changes in the viral antigenic structure which annually imposes the development of new vaccines, in particular, because of the emergence of the newly virulent viral strains. The time required for vaccine development has an impact on the hospitalization and mortality of vulnerable patients [13].

Cough medications, both expectorants (for treatment of productive cough) and antitussives (for treatment of dry cough), are frequently used; however, they will be discussed separately.

Furthermore, alternative herbal therapies have always been used for the prevention and treatment of common cold diseases and have been valued scientifically [14]. Several medicinal plants are commonly used as medicines to relieve the symptoms of upper tract respiratory infections such as relieving cough, reducing fever, and clearing the nose. Moreover, the treatment of colds depends mainly on compounds that are isolated from plants such as polyphenols, flavonoids, saponins, and alkaloids [15]. Different randomized, placebo-controlled trials indicated diverse herbs’ efficiency, for example, *Hedera helix* L. (*H. helix*) [16], *Thymus vulgaris* L. (*T. vulgaris*) [17], *Sambucus nigra* L. (*S. nigra*) [18], *Camellia sinensis* L. (*C. sinensis*) [19] and *Pelargonium sidoides* L. (*P. sidoides*) [20] for the treatment of common colds. However, the combination of several herbs such as *H. helix/Primula veris* L. (*P. veris*)/*T. vulgaris*) [21,22], essential oils [23] or *Echinacea purpurea* L. (*E. purpurea*) [24] are approved in several countries as medicinal products recommended for treatment of cold symptoms. This review presents an overview of the main herbs used as a treatment for common colds worldwide.

**Table 1:** Herbal drugs used for the relief of early symptoms of common cold/for the short-term prevention and treatment of common cold/for the relief of the symptoms of common cold/cough associated with cold/as an expectorant in cough associated with cold/with cold/for the relief of fever associated with common cold, with clinical trials or without [25].

Plant ^a^	Plant Part, Herbal Drug ^a^	Main Constituents ^b^
*Allium sativum* L.	bulb, *Allii sativi* bulbus	sulfoxide (alliin), steroidal saponins, flavonoids, *N*-feruloyltyramine, amino acids, adenosine
*Echinacea angustifolia* DC.	root, *Echinaceae angustifoliae* radix	phenylethanoids, chlorogenic acid, alkamides, polysaccharides, essential oil, glycoproteins, tussilagine, isotussilagine (traces)
*Echinacea pallida* (Nutt.) Nutt.	root, *Echinaceae pallidae* radix	phenylethanoids, chlorogenic acid, alkamides, polysaccharides, essential oil, glycoproteins
*Echinacea purpurea* (L.) Moench	root, *Echinaceae purpureae* radix; fresh aerial part, *Echinaceae purpureae* herba recens	phenylethanoids, chlorogenic acid, alkamides, polysaccharides, essential oil, glycoproteins, tussilagine, isotussilagine
*Eucalyptus globulus* Labill.	essential oil, *Eucalypti aetheroleum*; leaf, *Eucalypti folium*	main components: 1,8-cineole, limonene + hydroxycinnamic derivates, flavonoids, triterpenes, euglobals, macrocarpals, essential oil
*Filipendula ulmaria* (L.) Maxim.	flower, *Filipendulae ulmariae* flos; aerial parts, *Filipendulae ulmariae* herba	essential oil (main component: salicylaldehyde) flavonoids, tannins, phenolic glycosides, essential oil
*Foeniculum vulgare* Miller subsp. *vulgare* var. *vulgare*	essential oil, *Foeniculi amari* fructus *aetheroleum*; fruits, *Foeniculi amari* fructus	main components: *trans*-anethole, fenchone, limonene, *cis*-anethole + essential oil, glucosides of hemiterpenoids, monoterpenoids, fatty oil
*Grindelia robusta* Nutt. *Grindelia squarrosa* (Pursh) Dunal *Grindelia humilis* Hook. et Arn. *Grindelia camporum* Greene	aerial part, *Grindeliae herba*	resin, tannins, triterpenic saponins, flavonoids, phenolic acids, essential oil
*Glycyrrhiza glabra* L. *Glycyrrhiza inflata* Bat. *Glycyrrhiza uralensis* Fisch.	root, *Liquitiae* radix	triterpenic saponins, flavonoids, chalcones, coumarins, polysaccharides, sterols
*Marrubium vulgare* L.	aerial part, *Marrubii* herba	diterpenes, flavonoids, essential oil, betonicine, choline
*Matricaria recutita* L.	flower, *Matricariae* flos	flavonoids, essential oil, coumarins, *N*^1^,*N*^5^,*N*^10^,*N*^14^-tetra-*p*-coumaroylspermine, polysaccharides
*Mentha* × *piperita* L.	essential oil, *Menthae piperitae aetheroleum*	main components: menthol, menthone, menthylacetate, isomenthone, 1,8-cineole, menthofurane, limonene, pulegone, carvone, isopulegol
*Origanum dictamnus* L.	aerial part, *Origani dictamni* herba	essential oil, flavonoids, triterpenoids
*Pelargonium sidoides* DC *Pelargonium reniforme* Curt.	root, Pelargonii radix	coumarins, polyphenols, gallic acid derivatives
*Pimpinella anisum* L.	essential oil, *Anisi aetheroleum*; fruit, *Anisi* fructus	main components: *trans*-anethol, *cis*-anethol, linalool, *α*-terpineol + essential oil, flavonoids, fatty oil, coumarins
*Polygonium aviculare* L.	aerial part, *Polygoni avicularis* herba	flavonoids, coumarins, naphtoquinones, polysaccharides
*Polypodium vulgare* L.	rhizome, *Polypodii* rhizoma	steroidal saponins, triterpenoids, fatty oil, essential oil
*Primula veris* L. *Primula elatior* (L.) Hill	flower, *Primulae* flos; root, *Primulae* radix	flavonoids, saponins, carotenoids, volemitol, primine
*Salix purpurea* L. *Salix daphnoides* Vill. *Salix fragilis* L.	bark, *Salicis* cortex	phenolic glycosides, flavonoids, tannins (proanthocyanidins)
*Sambucus nigra* L.	flower, *Sambuci* flos	flavonoids, essential oil, spermidine, tannins, triterpenic acids, mucilage
*Sideritis scardica* Griseb. *Sideritis clandestina* (Bory and Chaub.) Hayek *Sideritis raeseri* Boiss. and Heldr. *Sideritis syriaca* L.	aerial part, *Sideritis* herba	essential oil, flavonoids, phenylethanoids, diterpenes
*Thymus vulgaris* L. *Thymus zygis* L.	essential oil, *Thymi* typo *thymolo aetheroleum* aerial part, *Thymi* herba	main components: thymol, linalool, carvacrol, α-terpinene, 4-terpineol + essential oil, flavonoids, monoterpenoid glucosides, hydroxycinnamic derivatives, polysaccharides
*Tilia cordata* Miller *Tilia platyphyllos* Scop. *Tilia* × *vulgaris* Heyne	flower, *Tiliae* flos	essential oil, flavonoids, tannins, mucilage, alkaloids (traces)
*Verbascum thapsum* L. *Verbascum densiflorum* Bertol. *Verbascum phlomoides* L.	flower, *Verbasci* flos	mucilage, flavonoids, phenylethanoids, triterpenic saponins, iridoids

^a^ botanical terminology as used in European Pharmacopoeia 10.7. [European Pharmacopoeia 10.7. Available online: https://pheur.edqm.eu/app/10-7/search?chapter=75351&title=12%20Herbal%20drugs%20and%20herbal%20drug%20preparations (accessed on 4 July 2022)];.^b^ Main constituents by Nagy et al. [26].

## 2. Possible Mechanism of Action of Phytochemicals for Colds

**Figure 1:**
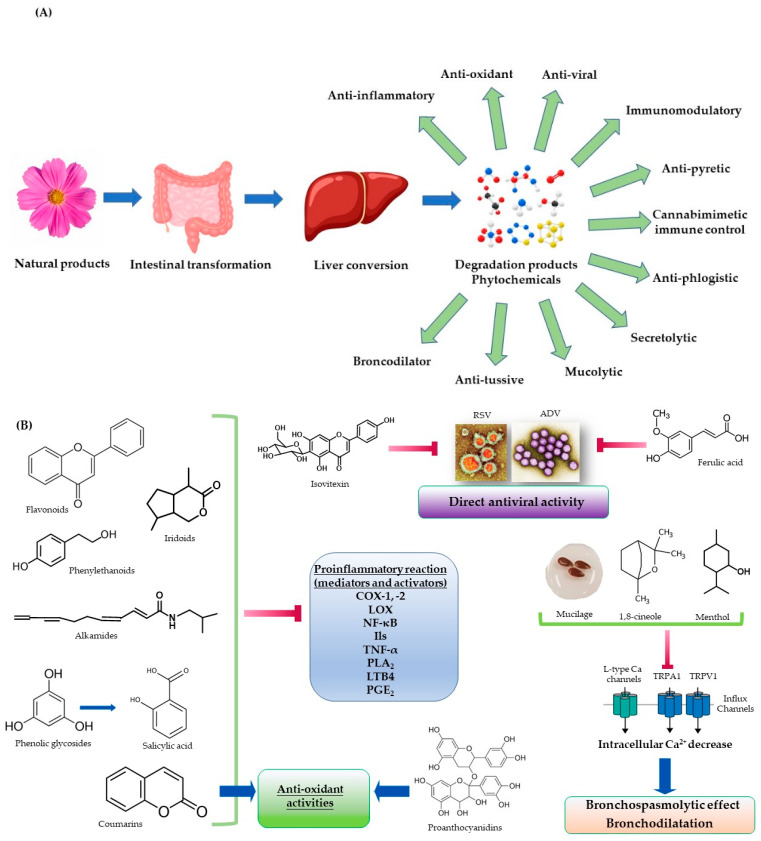
Mechanisms of the action of phytochemicals in the common cold. (**A**) The oral administration of plant products leads to their intestinal transformation, absorption, and conversion by the liver to degradation products, exhibiting different effects on common colds. (**B**) A number of phytochemicals such as flavonoids, phenylethanoloids, iridoids, and alkamides, and constituents of essential oils such as monoterpenoids, and phenolic glycosides inhibit the synthesis and the activity of different pro-inflammatory mediators. This effect includes the inhibition of the activities of cyclooxygenase-1, and -2 (COX-1, COX-2), lipoxygenase 5 and 15 (5-LOX, 15-LOX), transcription factors as NF-kB, interleukins 1β, 2, 6, and 8 (IL-1β, IL-2, Il-6, Il-8), tumor necrosis factor α (TNF-α), phospholipase A_2_ (PLA_2_), leukotriene B4 (LTB4), and prostaglandin E_2_ (PGE_2_). Other phytochemicals such as as coumarins and proanthocyanidins exhibit anti-oxidant activities, while compounds such as isovitexin and ferulic acid present direct antiviral activities against respiratory syncytial virus (RSV) and adenovirus (ADV), respectively. Mucilages and monoterpenoids can also exert bronchospasmolytic activities by calcium influx blockage through the inhibition of transient receptor potential ankyrin 1 (TRPA1) and transient receptor potential vanilloid 1 (TRPV1), or by blocking the L-type calcium channels, respectively.

Regarding the potential action of plants, their main active compounds have anti-inflammatory activity [26]. The degradation products (300–400 kDa) of acidic heteropolysaccharides, containing glucogalacturonic, galactolacturonic, and rhamnogalacturonic moiety [27] exhibit this effect. The flavonoids [28] involve the inhibition of the synthesis of different pro-inflammatory mediators such as eicosanoids, cytokines, histamine, and C-reactive protein. Activities of flavonoids include inhibition of phospholipase A_2_ (PLA_2_), cyclooxygenase-1 (COX-1), and lipoxygenase (LOX), as well as inhibition of tumor necrosis factor-α (TNF-α), interleukin-1β (IL-1β), IL-6, transcription factors such as nuclear factor-kappa B (NF-κB), activating protein-1 (AP-1) and nuclear factor erythroid 2-related factor 2 (Nrf2), decreasing inducible nitric oxide synthase (iNOS) activity, too [28]. The same activity has been cited for phenylethanoids (as verbascoside) [29]. The oligomeric proanthocyanidins [30] have an effect on the formalin-induced paw edema model of inflammation in rats, as well as are inhibitors of PLA_2_, COX-1, COX-2, 5-LOX, and 15-LOX; consecutively, the level of pro-inflammatory mediators (IL-1β, IL-2, IL-6, IL-8) decreased, through the inhibition of nitric oxide (NO) production and the suppression of NF-κB pathway activation. Proanthocyanidins also have anti-oxidant activities (with flavonoids) [30]. In addition, the natural compounds coumarins [31] are considered as an antioxidants. They have also an inhibitory effect on COX-1, 5-LOX, NF-κB, TNF-α, and NO production. The anti-inflammatory effects of iridoids (such as the hydrolyzed derivatives of aucubin, geniposide, genciopikrine, harpagide, catalpol, and loganin) [32] are attributed to the inhibition of COX-1 and COX-2, consecutively, the production of cytokines, PGE_2_, LTB_4_, TBX_2_, and IFN-γ. These effects reduce the expression of some proinflammatory cytokines, e.g., TNF-α, IL-1β, IL-2, and IL-6 [32]. Treatment with *Echinacea* causes a significant increase in neutrophils, monocytes, and leukocytes (immunostimulatory/immunomodulatory effect). The anti-inflammatory effect may be attributed to the inhibition of COX-1 and to a lesser extent of COX-2 by alkamides [33,34]. Additionally, it inhibited the production of NO and the fibroblast-induced collagen concentration/level. Alkamides also inhibit NF-κB, described as stimulating the expression of some mediators of inflammation, cytokines, e.g., TNF-α, IL-2, and IL-8. Alkamides [35] have cannabimimetic properties on both cannabinoid receptors (CB_1_ and CB_2_). The activation of the former plays a considerable role in controlling anxiety, and the latter is mainly involved in immune system activities (Figure 1A,B).

When taken orally, the Salicis cortex, which contains phenolic glycosides (salicin, salicortin, tremulacin), the glycosides undergo intestinal transformation to saligenin, which is absorbed and converted by the liver to salicylic acid [36]. Salicylic acid has recognized antipyretic and anti-inflammatory properties [36]. Constituents of essential oils such as monoterpenoids (borneol, 1,8-cineole, citronellal, fenchone, carvacrol, carvone, linalool, menthol, menthone, 4-terpineol, α-terpineol, thymol) and phenylpropanoids (anethole, cinnamic aldehyde, eugenol) [37,38] are also inhibitors of pro-inflammatory mediators such as leukotriene B4 (LTB4), prostaglandin E_2_ (PGE_2_), IL-1β, TNF-α, thromboxane B_2_ (TXB_2_) across the inhibition of COX-1, COX-2, NF-κB [37,38] (Figure 1B).

Other natural cold remedies are caffeic, ferulic, and chlorogenic acid [39,40,41], which have the antiphlogistic effect. This effect is explained by the inhibition of both NF-κB and by increasing the production of NO and PGE_2_; ferulic acid inhibits TNFα, and chlorogenic acid reduces IL-1β, IL-6, and TNFα production [39,40,41].

Cough medications, both expectorants (productive cough) and antitussives (dry cough), are frequently used [26]. They reduce the viscosity of tenacious secretions by irritating the gastric vagal receptors that stimulate respiratory tract fluid, thus increasing the volume but decreasing the viscosity of respiratory tract secretions [26].

The bronchodilator effect of the low concentration of volatile compounds (monoterpenes) as menthol (a TRPM8 agonist) and 1,8-cineole are associated with the calcium influx blockade through L-type calcium channels [42] (Figure 1B). Thymol and carvacrol are β_2_-agonists, and after decreasing intracellular calcium levels, they possess a bronchospasmolytic effect. Fenchone, 1,8-cineole, linalylacetate, citronellal, and α-terpineol have mucolytic activity. Labdane diterpene such as marrubenol (the substance of bitter taste receptors—T2Rs) inhibits bronchoconstriction by blocking L-type calcium channels [42]. Triterpenic saponins (as hederacoside C and B, and after their degradation, α-hederin, β-hederin, respectively; glycyrrhizine) [43] have β_2_-sympathomimetic activity by increasing secretion of pulmonary surfactant in lung alveolar type II cells, which could explain their secretolytic activity. Simultaneously, the level of intracellular calcium is decreased, which raises bronchodilatation (Figure 1B).

Mucilage is an active compound as an antitussive [26]. It inhibits the cough reflex suppressing the response of the cough center, via the demulcent effect of afferent vagus nerves. The blockade of transient receptor potential ankyrin 1 (TRPA1) and transient receptor potential vanilloid 1 (TRPV1) by mucilage represents a therapy for the treatment of dry cough. The TRPA1 agonists with antitussive activity are monoterpenes as borneol, 1,8-cineole, eugenol, menthol, thymol, and camphor (all in low concentrations) [42] (Figure 1A,B). 

Moreover, some plant compounds proved to have antiviral activity. Isovitexin and isoorientin (flavone 6-C-glucosides) [44] were tested for their antiviral activity against the respiratory syncytial virus. Phenolic compounds such as caffeic acid, *p*-coumaric acid, ferulic acid, and chlorogenic acid [45]; flavonoids such as quercetin [46] and apigenin; and triterpenes such as ursolic acid [47] have anti-adenoviral activity (Figure 1B).

## 3. Most Commonly Used Medicinal Plants for the Treatment of the Common Colds

To meet our objective, which is the use of natural herbal medicine to treat the common cold, we conducted a scientific bibliographic search of articles published from the year 2000 onwards in the following databases: Medline, PubMed, and Scopus. We used the following keywords: “plant”, “treatment”, and “cold” (Table 2 and Table 3).

### 3.1. Allium sativum *L.*

Garlic *Allium sativum* L. (*A. sativum*) has been widely known for centuries to influence health and to provide benefits to almost all physiologic systems including immunity. The antioxidant properties of *A. sativum* have also been also studied for their impact on human health, especially against tissue damage and inflammation [48]. Garlic is considered an antimicrobial and antiviral product that could relieve common cold viruses such as the human rhinovirus. A bibliometric study was performed to determine the effect of *A. sativum* on either the prevention or treatment of the common cold. However, data are limited to one clinical trial. The trial reported 24 occurrences of the common cold in the garlic intervention group (allicin content) compared with 65 in the placebo group (*p* < 0.001), resulting in fewer days of illness in the garlic group compared with the placebo group (111 versus 366). The authors of the clinical trial suggest that using garlic as a supplement to allicin can prevent the onset of common cold infections. However, the clinical data published in the bibliometric study were insufficient to validate the effect of garlic in the prevention or treatment of the common cold [49,50].

### 3.2. Echinacea angustifolia *DC.*, Echinacea pallida *(Nutt.) Nutt.*, Echinacea purpurea (*L.*) Moench

The medicinal herbal echinacea species have clinical properties essentially as an immune-modulator in the case of common colds and respiratory infections [51]. The immunological effects of echinacea have been investigated extensively in vitro and in vivo. Echinacea products are made from the roots, the whole plant, or aerial parts of *Echinacea angustifolia* (*E. angustifolia*), *Echinacea pallida* (*E. pallida*), or *Echinacea purpurea* (*E. purpurea*) [51,52].

The *E. purpurea* has been recommended as a medicinal plant for the treatment of the common cold, cough, bronchitis and upper respiratory infections [53]. In vitro experiments with human macrophages found that fresh-pressed juice and dried juice from the aerial parts of *E. purpurea* stimulated the production of cytokines including IL-1, IL-10, and TNF-α [54]. In addition, evidence pointed out the effective activity of *E. purpurea* towards Coronavirus disease 2019 (COVID-19) in an organotypic cell culture model [55,56,57].

The effectiveness of *E. purpurea* against colds was investigated by a randomized clinical trial performed on a total of 79 healthy children, designed to treat upcoming cold episodes with either 3 or 5 Echinaforce Junior tablets (EFJ) (contains the same herbal extract of freshly harvested *E. purpurea*) daily for up to 10 days. A total of 130 cold episodes were reported in 68 children overall during the 5.3 months of observation, encompassing a full cold and flu season. Results showed the treatment with EFJ had an excellent safety profile for the acute treatment of common cold symptoms [58]. In addition, in a randomized, double-blind, placebo-controlled trial, 282 subjects aged 18–65 years with a history of 2 or more colds in the previous year were recruited to evaluate the benefits of the treatment against cold symptoms. Among people who developed a cold, a significant decrease in symptoms was observed in the group that was under *Echinacea* treatment compared to the placebo group [59]. These findings confirmed the results of a randomized, double-blind, placebo-controlled clinical trial realized on 80 volunteers who had experienced the first symptoms of a cold. After a period of treatment with *E. purpurea* herb, this plant remains clinically efficient to relieve symptoms faster than a placebo [60]. However, *Echinacea* therapy represents an effective alternative to standard symptomatic medications in the acute treatment of the common cold [61].

### 3.3. Eucalyptus globulus *Labill.*

Evidence has been shown that a selection of natural herbs improves the relief of symptoms of upper respiratory tract infections and that their inhalation has antitussive and expectorant effects [62].

The genus *Eucalyptus* encompasses more than 900 species and subspecies. Different species of *Eucalyptus* are used in alternative medicine as antiseptics due to their antimicrobial and antioxidant properties to treat the common cold and respiratory infections [63,64,65]. In addition, a recent study was able to determine that a formula containing hyaluronic acids, *Eucalyptus* oil, copper, and manganese salts is effective for safe nasal irrigation and therefore for alleviating symptoms associated with the common cold such as nasal congestion [66]. Additionally, the *Eucalyptus* plant is often used as a compound in an ointment containing camphor, menthol, and *Eucalyptus* oils for topical application, which lessens the symptoms of colds [67,68]. A meta-analysis published in 2022 was able to synthesize the clinical trials that demonstrate the effectiveness of *Eucalyptus* in relieving cough. However, six studies have been published. Four randomized controlled trials have shown that *Eucalyptus globulus* Labill. (*E. globulus*) used in a formula significantly reduces cough compared to the placebo group. This study supports the idea of using the medicinal plant *Eucalyptus* as a remedy for cold symptoms primarily for cough and runny nose [69].

### 3.4. Grindelia robusta *Nutt.*/Grindelia squarrosa (*Pursh*) Dunal, Grindelia humilis *Hook. et Arn.*, Grindelia camporum *Greene*

EMA has pointed to the non-pharmaceutical value of several conger herbs in post-viral infection cough. The *Grindelia* plant is one of them [15]. However, it has been shown that *Grindelia squarrosa (G. squarrosa)* has an anti-inflammatory effect in inhibiting proinflammatory cytokines such as IL-8, TNF-α, IL-1β, and IL-6 in culture cells such as macrophage, neutrophil, and respiratory epithelial cell line [70,71]. The researchers suggest that the anti-cold activity of *Grindelia* comes down to the composition of the medicinal plant formula used, and that *G. squarrosa* could have an essential effect on the treatment of inflammatory diseases of the respiratory tract [70,71]. Moreover, *G. robusta* has been demonstrated to be effective as an expectorant, antitussive, and anti-inflammatory remedy in children, but no clinical trial has confirmed its efficiency [72].

### 3.5. Glycyrrhiza glabra *L.*, Glycyrrhiza inflata *Bat.*, Glycyrrhiza uralensis *Fisch*

The *Glycyrrhiza* plant is known for its anti-inflammatory and immunomodulatory properties during upper respiratory infections, targeting respiratory symptoms such as sore throat or cough. Its action is mediated by interfering with the Toll-like receptor 4 (TLR4) signaling and decreased production of pro-inflammatory cytokines including nuclear factor-κB, tumor necrosis factor, IL-1, and IL-6 [73].

The effect of *Glycyrrhiza* alone as an herbal remedy in the treatment of common colds is not yet clear, but several herbal preparations containing *Glycyrrhiza* have long been used to treat upper respiratory tract infections. In contrast, there are herb mixes containing *Glycyrrhiza*: Siji-kangbingdu, Maxing Shigan decoction, Lianhua-Qingwen capsules, and Macmoondongtang, which are used in China and Korea to treat upper respiratory tract infections [74,75].

A mixture of ASMATUSTM herbs has been used to relieve symptoms of asthma in children, and its activity has been evaluated as a remedy for colds. For example, a study has been conducted on 46 children with asthma and onset of cold symptoms. Patients received either the herbal mixture (composed of *Matricaria chamomilla* L., *Althaea officinalis* L., *Malva sylvestris* L., *Hyssopus officinalis* L., *Adiantum capillus-veneris* L., *G. glabra* and *Ziziphus jujube* Mills), or a placebo daily for 5 days. Indeed, the herbal mixture significantly decreased the severity of coughing and nocturnal awakenings compared to the placebo [76].

### 3.6. Mentha × piperita *L.*

*Mentha* × *piperita* L. (*M.* × *piperita*) is one of the herbs most widely used as a remedy for common colds [77,78]. The antiviral and anti-inflammatory activities of *M.* × *piperita* leaves have been investigated in the case of respiratory infection. In the in vitro model, *M. piperita* decreases the production of proinflammatory cytokines such as TNF-α, IL-6, and PGE2, and has antiviral activity against the respiratory syncytial virus (RSV) [79]. 

According to EMA, *M.* × *piperita* essential oil (inhalation) could be used as a treatment of cough and cold symptoms by stimulating cold receptors in the respiratory tract [80,81]

A prospective randomized double-blind controlled trial was assessed to investigate the effects of essential oils in patients with upper respiratory tract infections based on a sore throat, hoarseness, or cough. *Mentha* × *piperita* was investigated in an herbal mixture containing *E. citriodora, E. globulus*, *Origanum syriacum* L., and *Rosmarinus officinalis* L. A spray containing essential oils of plant mixture was applied five times a day for 3 days and compared with a placebo spray. Spray application reported an immediate improvement in the symptoms of upper respiratory ailments [82].

### 3.7. Origanum dictamnus *L.*

As noted in the previous section, *Origanum dictamnus* L. (*O. dictamnus)* was used among the herbal mixture that showed an effect on improving symptoms of upper respiratory infections [82,83]. EMA announced in its report related to this plant that the Committee on Herbal Medicinal Products stated that it can be used as a remedy for the symptoms of the common cold, but no clinical trial has been published to date [84].

### 3.8. Pelargonium sidoides *DC*, Pelargonium reniforme *Curt*

*Pelargonium sidoides* DC (*P. sidoides*) (family *Geraniaceae*) is a plant native to South Africa whose roots have been used as herbal remedies for respiratory and gastrointestinal infections for many centuries by the local South African populations [85]. A number of studies highlighted the activity of the proanthocyanidin-rich extract EPs 7630 from the roots of *P. sidoides.* It is considered effective against a variety of respiratory viruses such as HCoV (HCo-229E), influenza A virus (H1N1, H3N2), respiratory syncytial virus (RSV), and parainfluenza virus by limiting the symptom severity and disease duration of these infections [86,87].

A total of 120 patients with a common cold and at least 2 out of 10 common cold symptoms received one film-coated 20 mg tablet of EPs 7630 thrice daily for 10 days in an uncontrolled, interventional multicenter trial (ISRCTN65790556). Common-cold-associated symptoms and treatment satisfaction were evaluated after 5 days and at the treatment end. In 61 patients, viral nucleic acids were detected. Of these, 23 (37.7%) tested positive for at least 1 HCoV (HCoV subset) and 38 (62.3%) for other viruses only (non-HCoV subset). Patients of both subsets showed a significant improvement in common cold symptoms already after 5 days of treatment. EPs 7630 treatment outcomes of common cold patients with confirmed HCoV infection were as favorable as in patients with other viral infections [86]. Another clinical trial has evaluated the efficacy of EPs 7630, compared with the placebo for the treatment of the common cold. Of 207 patient participants, 103 were included in the standard dose of EPs 7630 (received 3 × 30 drops per day), 104 participated in the high dose group (received treatment with 3 × 60 drops per day), and the control group received a placebo. The study was conducted over a period of 10 days. The active EPs 7630 is an effective, well-tolerated, and safe treatment for the common cold. It significantly reduced the severity of symptoms [88]. The effectiveness of the extract roots of *P. sidoides* was also evaluated and confirmed by Lizogub [89] and Ross [90].

### 3.9. Pimpinella anisum *L.*

The Committee on Herbal Medicinal Products and EMA suggest that *Pimpinella anisum* L. *(P. anisum)* can be used as an expectorant (a medicine that helps bring up phlegm) for coughs associated with colds [91]. This evidence could be reinforced by the study of Iannarelli et al. [92]. The authors of this study evaluated the anti-inflammatory activity and the effect on mucin secretion of *P. anisum* essential oil in primary airway bronchial and tracheal epithelial cells (HBEpC and HTEpC, respectively). The data indicated that *P. anisum* essential oil showed a significant anti-inflammatory effect on both HBEpC and HTEpC cells together with mucus hypersecretion, which indicates that *P. anisum* could be used as an expectorant during cold periods [92]; however, on the other hand, there is no published clinical trial which confirms the expectorant effect of this plant. 

### 3.10. Primula elatior (*L.*) Hill, Primula veris *L.*

Official therapeutic indications are based on traditional use and include use as an expectorant in the cough due to a cold for both the preparations from flower and roots [93,94].

Parts of the plant used in official phytotherapy are flowers and/or roots. Flowers can be dried whole or cut and can or cannot include calyx [95]. The main constituents in flowers are triterpenic saponins, which are predominantly present in the sepals (up to 2%); flavonoids, which are predominantly present in the petals (up to 3%); carotenoids; essential oil (trace amounts); rosmarinic acid; d-volemitol; and other saccharide alcohols [95]. Roots may be whole or cut and dried [96]. The main constituents in roots are triterpenic saponins (3–12%), phenolic glycosides, saccharides, and d-volemitol (primulitol) [96].

According to the European Union herbal monograph, flowers are used as a herbal substance, comminuted and prepared as tea, or a liquid extract (prepared with 25% ethanol) [94,95]. The roots may be used as a herbal substance for the production of herbal preparations: dry extract (prepared with 40–50% ethanol), liquid extract (prepared with 70% ethanol), tincture (prepared with 70% ethanol), soft extract (prepared with water; 20–55% ethanol; 50% methanol; or a mixture of methanol, water, and ammonia), and comminuted herbal substance [93].

Since *Primula* flowers are usually used in combinations with other herbal substances (*Primula* content in teas is typically 10–30%, 1% in liquid, and 8% in solid dosage forms), assessing their effectiveness in the treatment of cough is challenging [95]. There are no clinical trials published in which only *Primula* flower or root preparation are used in the treatment group. However, combinations with thyme preparations show effectiveness on cough in clinical trials [97].

*Primula* preparations are generally safe to use, but their effectiveness in the treatment of cough is questionable. The mechanism of action for this indication is unclear and there is a lack of relevant clinical studies.

### 3.11. Sambucus nigra *L.*

*Sambucus nigra* or elderberry belongs to the family *Adoxaceae* [98].

The part of elderberry used in traditional and rational phytotherapy is the flower. Elderberry flowers are used dried and the herbal substance contains at least 0.80% flavonoids according to the European Pharmacopoeia [99]. Flavonoid content may be up to 3%. Other important compounds are triterpenes essential oil, 3% caffeic acid derivatives, 0.11% sterols, 8–9% minerals (especially potassium), and other (e.g., tannins, mucilages, pectins and saccharides, plastocynin) [99,100].

According to the European Union herbal monograph, elderberry flowers are used as an herbal substance and herbal preparations, i.e., comminuted, as a liquid extract (25% ethanol used as an extraction solvent) or as a tincture (25% ethanol used as extraction solvent). These can be further processed into tea or liquid oral dosage formulations. The EMA has approved the indication for “the relief of early symptoms of common cold”, which is based solely on traditional use [101].

In some cases, fruit is also used. Dried, ripe berries are the herbal substance, but fresh berries are also used in some preparations [102]. Fresh fruits contain anthocyanins and flavonoids. The dried seeds contain hemagglutinin (lectins, e.g., *S. nigra* agglutinin III), while *S. nigra* agglutinin Vf lectin is present in fresh fruits. Other constituents are essential oil (trace amounts), vitamins, minerals, and saccharides [102]. The seeds of the unripe fruits also contain cyanogenic glycosides, sambunigrin, prunasin, zierin, and holocalin [103]. Other traditional uses of elderberry fruit preparations are as a laxative, diaphoretic, diuretic, analgesic, and sedative, to alleviate headache, dental, heart, nerve pain and neuralgia, and against cough and cold, to name a few [102]. However, the EMA monograph on elderberry fruit is not available yet.

To conclude, the data from clinical studies and mechanistic studies are too scarce to unambiguously support the evidence-based use of elderberry preparations for the relief of the symptoms of the common cold. However, the use of flower preparations is safe and has an approved indication based on long-standing use. Elderberry fruit, although safe to consume if ripe, was not granted an indication by the EMA due to the lack of relevant data.

### 3.12. Sideritis scardica *Griseb.*/Sideritis clandestina *(Bory and Chaub.) Hayek.*/Sideritis raeseri *Boiss./and Heldr.*, Sideritis syriaca *L.*

The Committee on Herbal Medicinal Products and EMA reported that the three species are used as a remedy for cold symptoms and primarily for cough prevention in Albania, Greece, and Bulgaria [104]. Other evidence noted that *Sideritis scardica* Griseb (*S. scardica)* has been used in traditional medicine as a remedy for improving symptoms during respiratory infections such as bronchitis and bronchial asthma. Additionally, this plant is known for its effectiveness against colds and coughs [105,106], but there is no published clinical trial which confirms these effects.

### 3.13. Thymus vulgaris *L.*, Thymus zygis *L.*

The genus *Thymus* or thyme (family *Lamiaceae*) numbers about 400 species. This species is a common perennial shrub, native to southern Europe and the western Mediterranean.

Thyme is a traditional Mediterranean aromatic plant used for centuries in cooking, medicine, and perfume preparations. Consequently, thyme has spread worldwide, as has its uses, and this has led to many varieties. Nowadays, thyme cultivation is economically important to producing essential oils for the perfume industry, monoterpene production, and medicinal use. Thyme could also be used as a preservative for food products. Thyme leaves are also used as a fresh or dry condiment. The EMA published two monographs about the use of thyme for cough and cold, one for the herb and the second for the essential oil. The herbal substances and the herbal preparation(s) are composed of the leaves and/or flowers separated from the dried stems of the plant. The essential oil is obtained by steam distillation from fresh flowering aerial parts of *T. vulgare* or *T. zygis* [107,108].

As a member of the *Lamiaceae* family, essential oil production by thyme is important [109,110,111] To date, six main chemotypes have been described with main constituents: [109,110,112]: chemotype geraniol, terpineol, thuyanol-4, linalool, carvacrol, and thymol. EMA herbal monograph lists 10 chemotypes, underlying the chemical diversity of this plant [107].

In the recent article by Silva et al. on phenolic acids, tannins, rosmarinic and salvianolic acid, flavonoids, and polyphenols also enter frequently the chemical composition of *Thymus*.

Its therapeutic properties derive mainly from the essential oil with antitussive, expectorant, antiseptic, antimicrobial, and anthelmintic properties. Traditional uses also report hepatoprotective properties. No genotoxicity has been reported. However, considering the growth area of the plant, it could be contaminated with pollutants such as trace metals such as lead (Pb). Additionally, the chemical stability of essential oils is low [109]. Flavonoids from thyme do not seem to have a strong pharmacological potential [109].

Leaves are mostly used as an infusion, or as tea, ointment, and syrup. The reported properties of thyme infusion are as a general stimulant, anti-flatulent, cough depressant, common cold treatment, and antibacterial [109,111,113]. The anti-microbial properties are used in mouth, gastrointestinal, and urinary tract infections, as well as respiratory diseases (cough, bronchitis, asthma). The most important medicinal use relies on thymol, *p*-cymene, and carvacrol. 

Almost all medicinal applications are due to the antioxidant, anti-inflammatory (anti-TNFα et IL-1β), and antimicrobial activities (antibacterial including antibiofilm activity, fungicidal, virucidal) [17,112,113,114,115,116,117]. Kowalsky et al. suggested that the use of thymol in combination with an antibiotic may lead to reduced antibiotic concentration in therapeutic use against planktonic and biofilm-forming bacteria [17].

It is suggested that thymol antiviral activity may occur at a different level of the viral cycle, from cell penetration and viral envelope interference to protein inhibition [17]. 

Boskabady et al. [118] described a relaxant effect on the tracheal chain on guinea pigs as efficient as theophylline, and Wienkotter et al. [119] described a β_2_ agonist activity (molecular binding and biological effects), suggesting an interesting therapeutic activity at both symptomatic and viral levels in the case of upper respiratory tract infections such as the common cold and flu. Additional anti-inflammatory activities lead *Thymus* sp. to have strong potential in upper respiratory tract viral infections. Patil et al. [111] reported an inhibition of the 5-lipooxygenase at a low concentration of thyme essential oil (IC_50_ at 0.005 µg/mL), suggesting the latter could reduce leukotrienes synthesis during inflammation. 

Lenz et al. [120] also showed antiviral activity of thyme extract (in a commercial preparation, where the active compound is thymol according to the authors) against the human rhinovirus 1a (HRV1a, MDCK/A549 cells, MOI 1).

From a clinical point of view, Kemmerich et al. [22] published a clinical trial (doubled blind, placebo controlled, multicenter, with 363 patients including 182 with herbal treatment and 181 in the placebo group) which showed the efficacy and tolerability of a fluid extract of thyme herb (mixed with Ivy) on acute bronchitis. The herbal treatment significantly reduced cough compared to placebo (77.6% vs. 55.9%; *p* < 0.0001) after 9 days and reduced the severity of bronchitis (evaluated with BSS score). The authors also reported the superiority of the herbal treatment on sleep disturbance, and general well-being. They also reported that adverse effects were low and similar to those in the placebo group (3.8% and 4.5% respectively). All adverse effects had been resolved at the end of the study and the authors concluded the very good tolerability of the treatment [22].

Thymol is the most studied essential oil constituent from thyme and show good activity against several viruses (rhinoviruses, influenza viruses) in vitro, in vivo, and in clinical trials. The latter also shows the efficiency as well as the safety of thyme extracts in symptom reduction. The mechanism of action is not yet fully understood. However, several studies showed that thyme could reduce inflammation, enhance bronchodilatation via β2 agonism, and directly reduce viral replication. Thus, finally, thyme showed a direct and indirect strong potential in upper viral tract infections [121,122].

According to https://clinicaltrials.gov (accessed on 21 September 2022) and Silva et al. (2021), two clinical studies are still undergoing so as to better evaluate thyme, thyme extracts, or essential oils in upper respiratory tracts infections (NCT03218696 [123], NCT02981147 [109,124]).

To conclude, regarding the safe traditional use of thyme herbal preparation and essential oils in productive cough and common cold associated with in vitro, in vivo, and clinical data, common thyme is safe and efficient in this indication, as mentioned in the revised final opinion of the EMA.

### 3.14. Tilia cordata *Miller*, Tilia platyphyllos *Scop.*, Tilia × vulgaris *Heyne*

In an assessment report relating to EMA and the Committee on Herbal Medicinal Products, it was reported that the plants *Tilia cordata* Miller (*T. cordata*), *Tilia platyphyllos* Scop (*T. platyphyllos*), and *Tilia* × *vulgaris* Heyne (*T. vulgaris)* could be used as a remedy for cold symptoms in Germany and Poland in association with other plants such as *Salicis cortex*, *Thymi herba*, *Foeniculi amari fructus* and *Lichen islandicus* [125].

### 3.15. Verbascum thapsum *L.*, Verbascum densiflorum *Bertol.*, Verbascum phlomoides *L.*

*Verbascum* spp. is one of the natural botanical remedies used to alleviate cold-related symptoms [126]. It is known to relieve respiratory system ailments such as hoarseness, tonsillitis, colds, coughs, asthma, or bronchitis, and this data are supported by EMA and Committee on Herbal Medicinal Products [127,128].

Experimental studies have demonstrated the anti-inflammatory effect of the plant *Verbascum thapsum* L. (*V. thapsum)* by the fact that it has the ability to reduce the secretion of proinflammatory mediators in human myelomonocytic leukemia cells [129] and by its antiviral activity against the H1N1 infection in a mouse model [130].

## 4. Medicinal Plants without EMA Monography with Potential Effects on Common Cold

### 4.1. Aloe qrborescens *Mill.*

*Aloe arborescens* Mill. (*A. arborescens*) has been used mostly in the treatment of upper respiratory tract infections. Preclinical and clinical data showed that this medicinal plant has immunomodulatory and antiviral effects in the case of upper respiratory tract infections [135,136]. *A. arborescens* extract mixed with Vitamin C (1920 mg of the extract and 51 mg Vitamin C per 5 mL) “Biaron C^®^” has been tested against a panel of viruses that cause upper respiratory infections [137]. Human rhinovirus B (HRV14), influenza A virus (H1N1) and (H3N2), influenza B, respiratory syncytial virus (RSV), parainfluenza type 3 virus (Para 3), coxsackievirus (CA9), adenovirus C (Adeno 5) were cultivated in specific cells (such as MDCK, Hep-2, and HeLa-cells), and were treated by Biaron C^®^ at 2–0.006% of final concentration. Data demonstrated that the Biaron C^®^ was effective against the RNA viruses tested. The antiviral activity was more selective against Picornaviridae and Orthomyxoviridae. This confirms its known therapeutic antiviral effect [137].

### 4.2. Boehmeria jamaicensis *Urb.*

The extract of *Boehmeria jamaicensis* Urb. (*B. jamaicensis*) has been evaluated in the treatment of the common cold. Williams *et al*. tested the effect of *B. jamaicensis* extract on lymphocytes obtained from patients suffering from the common cold after four days of infection. However, the authors demonstrated that after the pretreatment of lymphocytes at a concentration of 25 µg/mL, interferon (IFN-gamma) was increased by 24.03 fold and interleukin 4 (IL-4) by 1.71 fold compared to the healthy group [138].

### 4.3. Camellia sinensis (*L.*) *Kuntze*, Camellia qssamica var. Kucha

Tea is a vegetable infusion containing antioxidants and other beneficial nutrients such as L-theanine (known to stimulate human T lymphocytes to secrete IFN-γ), a powerful antimicrobial cytokine. There are hundreds of varieties of the tea species such as *Camellia sinensis* (*C. sinensis*). Numerous observational studies suggest that tea drinking is beneficial to health. A randomized, double-blinded study demonstrated the effect of *C. sinensis* (as green tea) on cold and flu symptoms by enhancing immunity response [139]. The results of this study show that the ingestion of two capsules containing *C. sinensis* daily decreased by about a third the number of subjects who presented cold and flu symptoms. In addition to its effect on the incidence of cold and flu symptoms, *C. sinensis* ingestion enhanced the production of IFN-γ by γ/δ T cells challenged in vitro [139]. 

Compounds isolated from tea were evaluated for their cytotoxicity and inhibitory effects on human influenza virus A/Puerto Rico/8/34 by analyzing viral protein expression and progeny production. The concentration of theacrine or strictinin for effective inhibition of the influenza virus is approximately 50 μM [140].

### 4.4. Cistus × incanus. *L.*

The *Cistus incanus* (*C. incanus*) is a shrub of the *Cistaceae* family, a hybrid between *C. albidus* and *C. crispus*, evergreen which gives gray-green leaves and beautiful pink-purple flowers. It is mainly found in arid Mediterranean regions with soils rich in magnesium. This plant is the richest in polyphenols (antioxidants), and it is used in particular for its powerful antiviral and antibacterial properties [141,142].

The leaves of this plant (family *Cistaceae*) are traditionally used. It was reported that a *Cistus extract* (CYSTUS052) was more effective than an extract of green tea against infections of the upper respiratory tract [143]. Furthermore, in the case of the latter infections, a randomized, placebo-controlled clinical study was performed to evaluate the effect of a *Cistus extract* (CYSTUS052) in a total of 160 patients by comparing symptoms and by dosing inflammatory markers in either group: CYSTUS052 treatment and placebo. However, common cold symptoms and C-reactive protein were significantly reduced in CYSTUS052 treated group compared to the placebo group [144]. In addition, the antiviral activity of this extract (CYSTUS052) was determined in vitro and in vivo against the influenza A virus. Droebner et al. showed a major (≅ 90%) reduction in plaque numbers in CYSTUS052 pretreated cells in Madin–Darby canine kidney (MDCK) cells, as well as the absence of common cold symptoms in vivo in influenza A virus-infected mice treated with CYSTUS052 [145]. Ehrhardt et al. demonstrated the anti-influenza effect of plant extract CYSTUS052 in vitro on H5N1 infected adenocarcinoma human alveolar basal epithelial cells (A549) or MDCK cells. The effective dose of *Cistus extract* plant was 50 μg/mL [146]. 

### 4.5. Cinnamomum verum *J. Presl* (*syn.* Cinnamomum cassia *J. Presl*)

*Cinnamomum cassia* (*C. cassia*) is a common traditional Chinese herbal medicine that has been used in the case of different respiratory tract diseases including the common cold. Antiviral activity of the hot water extract of *C. cassia* against HRSV was evaluated against both human upper (HEp-2) and lower (A549) respiratory tract cell lines. This study demonstrates that Cinnamomum cassia at 10 μg/mL could inhibit HRSV-induced plaque formation on A549 and HEp-2 cells by inhibiting viral attachment and penetration. Furthermore, *C. cassia* could decrease the expression of viral F protein to inhibit viral spread into other cells. The authors suggest that *C. cassia* could be an antiviral agent used in therapeutic ways to manage HRSV infection [147].

### 4.6. Larix decidua *Mill.*

This tree from the family *Pinaceae* produces the Larch arabinogalactan possessing an anti-common cold effect [148]. Larch arabinogalactan seems to be implicated specifically in the activation of immune cells as well as the secretion of pro-inflammatory cytokines during common cold diseases [149]. This scientific evidence could be confirmed by a clinical trial that enrolled a total of 199 healthy participants who had common cold infections in the last 6 months. A group of 101 participants were given Larch arabinogalactan (4.5 g) compared to the placebo group (98 participants). Subsequently, the results revealed that Larch arabinogalactan decreased significantly the incidence of common cold infections and reduced the number of seasonal common cold episodes [150]. Larch arabinogalactan seems to be implicated specifically in the activation of immune cells as well as the secretion of pro-inflammatory cytokines during common cold diseases [149]. 

### 4.7. Paeonia lactiflora *Pall.*

*Paeonia lactiflora* (*P. lactiflora*) is a component of Sheng-Ma-Ge-Gen-Tang (SMGGT; Shoma-kakkon-to) and Ge-Gen-Tang (GGT; kakkon-to) traditional Chinese blend known to have an antiviral activity against the human respiratory syncytial virus (HRSV). The latter activity of *P. lactiflora* has been tested against both human upper (human larynx epidermoid carcinoma cell line, HEp-2) and lower (human lung carcinoma cell line, A549) respiratory tract cells. These experimentations demonstrated that *P. lactiflora* could effectively inhibit HRSV replication and stimulate antiviral cytokine production [151]. In addition, the antiviral activity of *P. lactiflora* was also evaluated against both HRSV-2 and HRSV-4 in MRC5 cells. Treatment of infected cells by 1,2,3,4,6-penta-O-galloyl-β- d-glucopyranose (PGG) *P. lactiflora* root extract induced an antiviral activity toward HRV-2 (17.89 μM) and HRV-4 (17.33 μM) in MRC5 cells by interfering with the entry of the viruses into the cells and by decreasing rhinovirus receptors and inflammatory immune response mediators [152].

## 5. Herbal Combinations Useful for Treating the Common Cold

### 5.1. Hedera helix/Primula vulgaris/Thymus vulgaris

*Thymus vulgaris* is indicated mostly for the treatment of respiratory diseases [118], and a double-blind, randomized study identified the efficacy and tolerability of ivy leaves for the treatment of acute bronchitis with improving symptoms: cough, sputum, rales/rhonchi, and chest pain during coughing and dyspnea [153]. A combination of thyme and ivy leaves has been studied in the case of acute bronchitis with productive cough [21,22]. The efficacy of combination of thyme and ivy treatment was evaluated by a double-blind, placebo-controlled, multicenter Phase IV study performed on 361 outpatients with acute bronchitis and coughing fits. Coughing fits intensity and the symptoms of acute bronchitis improved rapidly in the group under the thyme–ivy combination [22,119]. Metanalysis demonstrated that different herbal mixtures are effective against the common cold such as vy/primrose/thyme, essential oils GeloMyrtol^®,^ and Tsumura bakumondoto (Table S1) [23]. This study highlighted that the combination of ivy/primrose/thyme has been effective to reduce coughing. Furthermore, RCT demonstrated also the effectiveness of GeloMyrtol^®^ and Tsumura bakumondoto [23]. 

### 5.2. Tsumura Bakumondoto

A study performed on 19 patients compared the effects of a bakumondoto preparation in combination with conventional medication to conventional medication alone [154]. The effect of bakumondoto on the intensity of the patient’s coughs was significantly stronger on days 4 and 5 of intake. No serious adverse events were reported [154]. 

### 5.3. GeloMyrtol^®^ (Myrtol^®^) 

This is a mix of plant medicine syrup produced by distillation of essential oils (Table S1). Myrtol^®^ 300 mg was investigated as treatment in a double-blind, parallel-group enrolling about 413 patients with bronchitis. The patients responded positively to this treatment with an improvement in their clinical state of health [155]. Myrtol^®^ was also confirmed to be effective against acute bronchitis. A study by Mattys et al. studied the effectiveness of the myrtol stand on 676 patients with acute bronchitis. The responder rate was significantly higher in the myrtol stand group and superior in terms of efficacy compared to other tested treatments and placebo; it is even considered as an alternative to antibiotics for acute bronchitis treatment [156]. 

### 5.4. Soshiho-Tang

Soshiho-tang is a widely used Oriental herbal formulation for treating the common cold in East Asian countries (Table S1). According to the meta-analysis of Jung et al. [157], Soshiho-tang is mainly used in the treatment of pediatric cough, nasal congestion, and runny nose, and because of the herbs that compose it, there could be an anti-inflammatory effect [157]. A recent review suggests that Soshiho-tang could be used in the treatment of COVID-19 symptoms [158]. 

### 5.5. Kang Jang^®^

Kang Jang^®^ is an herbal mix constitute (Table S1) including harms root used in Scandinavia for the treatment of respiratory tract diseases [159]. Bath et al. facilitated a clinical trial to test the effectiveness of Kang Jang^®^ as an antitussive remedy. Among 177 patients enrolled in the parallel-group, randomized, double-blinded, placebo-controlled trial, the group which had undertaken Kang Jan (30 mL/day; 762 mg genuine extracts with standardized contents of 0.2 mg/mL vasicine, 0.8 mg/mL chicoric acid, and 0.03 mg/mL eleutherosides B and E) showed a faster symptom improvement compared to other groups (placebo and bromhexine) [159].

### 5.6. So-Cheong-Ryong-Tang and Yeon-Gyo-Pae-Dok-San

Byun et al. investigated the extracts of both medicinal herbal mix (Table S1), So-cheong-ryong-tang and Yeon-gyo-pae-dok-san in the treatment of the common cold [160]. In this study, 480 patients who presented symptoms of the common cold within 48 h were recruited. The clinical trial was double blinded, and So-cheong-ryong-tang and Yeon-gyo-pae-dok-san were administrated separately in parallel groups orally 3 times per day. Evaluation of symptoms after mix herbal intake showed that each of the herbal blends used resulted in their improvement [160].

### 5.7. Combination of Echinaceae radix, Baptisiae radix, Thujae herba

The efficacy of the herbal combination *Echinaceae radix*, *Baptisiae radix*, and *Thujae herba* was evaluated in a randomized double-blind placebo-controlled study. A total of 238 valid patients with common cold symptoms were included [24]. The results showed that more than half of the patients taking the herbal combination responded positively to the treatment compared to patients taking a placebo. The therapeutic benefit of the herbal remedy appeared on day 2 and reached significance on day 4, continuing until the end of treatment while following the evolution of the symptoms of bronchitis and rhinitis [24]. The efficacy of this herbal medication in common cold treatment was also demonstrated by Naser et al. [161]. The authors evaluated in their clinical trial the effect of the mixture of *Echinaceae radix*, *Baptisiae radix*, and *Thujae herba* on runny nose symptoms. A total of 60 patients suffering from the common cold were treated with the mixture (19.2 mg and 9.6 mg) versus 31 patients who received a placebo, three times daily for 3–12 days. Test evaluation of common cold symptoms showed that the two groups that took the two doses (high and low) of the herbal mixture responded well to the treatment compared to the placebo [161]. Thus, both studies showed that this herbal remedy is effective and safe in common cold treatment.

### 5.8. Ma-Xing-Shi-Gan-Tang

Ma-xing-shi-gan-tang is a Chinese herbal mix (Table S1) used for the treatment of the common cold, fever, and influenza virus infections. The antiviral activity of nontoxic concentrations of Ma-xing-shi-gan-tang against influenza virus A/WSN/33 (H1N1) was examined in MDCK cells. Ma-xing-shi-gan-tang exhibited an antiviral activity with EC_50_ of 0.83 ± 0.41 mg/mL against influenza virus A/WSN/33 (H1N1). In addition, this combination of medicinal herbs appeared to have an inhibitory effect on the synthesis of both viral RNA and proteins in treating cells. These data supported that Ma-xing-shi-gan-tang could be used in the treatment of influenza virus infections [162].

## 6. Conclusions

To date, there is no specific treatment for the common cold. The only treatments that exist are aimed at relieving cold symptoms such as cough, fever, and runny nose. The development of research on medicinal plants has demonstrated their effectiveness against respiratory infections and in particular the common cold. Various clinical trials have established herbal syrups or herbal combinations that can be used for self-medication to relieve certain cold-related symptoms. *A. sativum*, *Echinacea* family, and *T. zygis* are considered antimicrobial natural products that can relieve common cold. Further future studies are recommended to determine alternative herbal remedies to prevent and treat other respiratory infections (viral and bacterial), primarily those for which vaccines and treatments are not yet well developed.

The use of medicinal plants to alleviate cold symptoms would be very interesting because they are more easily accessible to patients than drugs and compliance is generally better.

## Figures and Tables

**Table 2:** Medicinal plants used in the treatment of the common cold.

Plant	Family	Infection	References
*Allium sativum* L.	*Amaryllidaceae*	Common cold; COVID-19; Rhinoviruses.	[48,49,50]
*Echinacea purpurea* L., *Echinacea angustifolia* DC.	*Asteraceae*	Common cold; Coronavirus 229E and SARS-CoV-2; Rhinovirus colds.	[51,52,53,54,55,56,57,58,59,60,61]
*Eucalyptus globulus* Labill.	*Myrtaceae*	Acute respiratory infection.	[62,63,64,65,66,67,68,69]
*Grindelia robusta* Nutt, *Grindelia squarrosa* (Pursh) Dunal, *Grindelia humilis* Hook. et Arn., *Grindelia camporum* Greene	*Asteraceae*	Acute respiratory infection.	[15,70,71,72]
*Glycyrrhiza glabra* L., *Glycyrrhiza inflata* Bat., *Glycyrrhiza uralensis* Fisch	*Fabaceae*	Upper respiratory infections; common colds.	[73,74,75,76]
Mentha × piperita L.	*Lamiaceae*	Common colds; respiratory syncytial virus (RSV).	[77,78,79,80,81,82]
*Origanum dictamnus* L.	*Lamiaceae*	Upper respiratory infections.	[82,83,84]
*Pelargonium sidoides* DC, *Pelargonium reniforme* Curt.	*Geraniaceae*	Common cold; Acute respiratory tract infections.	[85,86,87,88,89,90]
*Pimpinella anisum* L.	*Apiaceae*	Expectorant Common cold.	[91,92]
*Primula elatior* (L.) *Hill, Primula veris* L.	*Primulaceae*	Antitussive.	[93,94,95,96,97]
*Sambucus nigra* L.	*Adoxaceae*	Common cold and influenza (A and B).	[98,99,100,101,102,103]
*Sideritis scardica* Griseb./*Sideritis clandestina* (Bory and Chaub.) Hayek./*Sideritis raeseri* Boiss./and Heldr., *Sideritis syriaca* L.	*Lamiaceae*	Bronchitis; bronchial asthma;common colds.	[104,105,106]
*Thymus vulgaris* L., *Thymus zygis* L.	*Lamiaceae*	Antitussive; common cold; Human rhinovirus.	[17,22,107,108,109,110,111,112,113,114,115,116,117,118,119,120,121,122,123,124]
*Tilia cordata* Miller, *Tilia platyphyllos* Scop., *Tilia* × *vulgaris* Heyne	*Tiliaceae*	Common cold.	[125]
*Verbascum thapsum* L., *Verbascum densiflorum* Bertol., *Verbascum phlomoides* L.	*Scrophulariaceae*	Common colds; coughs; asthma; bronchit.	[126,127,128,129,130]
*Foeniculum vulgare* Miller subsp. *vulgare* var.	*Apiaceae*	Expectorant.	[131]
*Matricaria recutita* L.	*Asteraceae*	antitussive bronchitis; fever; colds.	[76,132]
*Polygonium aviculare* L./*Polypodium vulgare* L.	*Polygonaceae*	Common colds.	[133]
*Salix purpurea* L./*Salix daphnoides* Vill./*Salix fragilis* L.	*Salicaceae*	Common colds.	[134]
*Aloe arborescens* Mill.	*Asphodelaceae*	Upper respiratory tract infections; Human rhinovirus B (HRV14), influenza A virus (H1N1) and (H3N2), influenza B, respiratory syncytial virus (RSV), parainfluenza type 3 virus (Para 3).	[135,136,137]
*Boehmeria jamaicensis* Urb.	*Urticaceae*	Common colds.	[138]
*Camellia sinensis* (L.) Kuntze, *Camellia assamica var*. *kucha*	*Theaceae*	Anti-Influenza viral adsorption and suppressed replication Cold viruses Common cold.	[139,140]
*Cistus* × *incanus* L.	Cistus	Common colds; upper respiratory tract; Anti-Influenza.	[141,142,143,144,145,146]
*Cinnamomum verum J.S. Presl*	*Lauraceae*	Common cold. Chronic bronchitis Human respiratory syncytial virus.	[147]
*Larix decidua* Mill.	*Pinaceae*	Common cold.	[148,149,150]
*Paeonia lactiflora* Pall.; *Paeonia veitchii Lynch*	*Fabaceae*	Rhinoviruses.	[151,152]

**Table 3:** Herbal combinations used in the treatment of the common cold.

Plants	Infection	References
Ivy (Hedera helix)/primrose (Primula vulgaris)/thyme (Thymus vulgaris)	Common cold Acute bronchitis	[21,22,23,117,118,153]
Tsumura bakumondoto	Common cold	[154]
Gelo Myrtol^®^	Common cold	[155,156]
Soshiho-tang	Common cold (chills and fever) Pulmonary disease	[157,158]
Kan Jang^®^	Respiratory tract infection	[159]
So-cheong-ryong-tang Yeon-gyo-pae-dok-san	Common cold	[160]
*Echinaceae radix*, *Baptisiae radix*, and *Thujae herba*.	Acute viral respiratory tract infection	[24,161]
Ma-xing-shi-gan-tang:	Common cold, fever, and influenza virus infections	[162]

## Data Availability

Data sharing is not applicable.

## Supplementary Materials

The following supporting information can be downloaded at: https://www.mdpi.com/article/10.3390/ph16050662/s1, Chapter 1: Other medicinal plants with EMA monography with potential effects on common cold. Table S1: Herbal combinations used in the treatment of the common cold.
