# Supplementary material for: Natural Products for the Prevention and Treatment of Common Cold and Viral Respiratory Infections
Source: Pharmaceuticals (Basel). 2023 Apr 28;16(5):662. doi: 10.3390/ph16050662 (PMC10220542; doi:10.3390/ph16050662)
Supplement: Supplementary file 1 [file pharmaceuticals-16-00662-s001.zip › pharmaceuticals-2279266-supplementary.pdf]

*Supplementary Materials*

# Natural Products for the Prevention and Treatment of Common Cold and Viral Respiratory Infections

**Nour Mammari [1], Quentin Albert [2,3], Marc Devocelle [4,5], Maša Kenda [6], Nina Kočevar Glavač [6], Marija Sollner Dolenc [6], Laura Mercolini [7], Jaroslav Tóth [8], Nagy Milan [8], Szilvia Czigle [8], Mihayl Varbanov [1,9,*] and on behalf of the OEMONOM [†]**

[1] CNRS, L2CM, Université de Lorraine, 54000 Nancy, France
[2] INRAE, Aix Marseille Université, UMR1163 Biodiversité et Biotechnologies Fongiques, 13288 Marseille, France
[3] INRAE, Aix Marseille Université, CIRM-CF, 13288 Marseille, France
[4] SSPC (Synthesis & Solid State Pharmaceutical Centre), V94 T9PX Limerick, Ireland
[5] Department of Chemistry, Royal College of Surgeons in Ireland, RCSI University of Medicine and Health Sciences, 123 St. Stephen's Green, D02 YN77 Dublin, Ireland
[6] Faculty of Pharmacy, University of Ljubljana, Aškerčeva cesta 7, 1000 Ljubljana, Slovenia
[7] Research Group of Pharmaco-Toxicological Analysis (PTA Lab), Department of Pharmacy and Biotechnology (FaBiT), Alma Mater Studiorum—University of Bologna, Via Belmeloro 6, 40126 Bologna, Italy
[8] Department of Pharmacognosy and Botany, Faculty of Pharmacy, Comenius University Bratislava, Odbojárov 10, 832 32 Bratislava, Slovakia
[9] Laboratoire de Virologie, CHRU de Nancy Brabois, 54500 Vandœuvre-lès-Nancy, France
* Correspondence: mihayl.varbanov@univ-lorraine.fr; Tel.: +33-3-72-74-73-19
† Listed at the end of Acknowledgments.

## 1. Other medicinal plants with EMA monography with potential effects on common cold

### 1.1 *Foeniculum vulgare* Miller subsp. *vulgare* var. *vulgare*

According to the EMA, the herb *Foeniculum vulgare* Miller subsp. *vulgare* var. *vulgare* is used as an expectorant in coughs associated with colds. The expectorant effect of this plant is essentially due to its composition. Indeed, the two main components anethole and estragole are recognized by their traditional use of cough associated with colds [131].

### 1.2 *Matricaria recutita* L.

Is known to treat cough and bronchitis, fever, colds, inflammation and infection [132]. As in the case of the plant *Glycyrrhiza*, it has been used in the mixed herb ASMATUSTM to relieve the symptoms of colds in asthmatic children [76].

### 1.3 *Polygonium aviculare* L. / *Polypodium vulgare* L.

The Committee on Herbal Medicinal Products and EMA reported that *Polygonium aviculare* L. can be used for the relief of symptoms of a

common cold, and for treating symptoms of minor mouth or throat inflammation [133].

*1.4 Salix purpurea* L. / *Salix daphnoides* Vill. / *Salix fragilis* L.

The Committee on Herbal Medicinal Products and EMA reported that the traditional herbal *Salix purpurea* L. / *Salix daphnoides* Vill. / *Salix fragilis* L. product used for the relief of fever associated with the common cold [134].

**Table S1.** Herbal combinations used in the treatment of the common cold.

| Plant | *Family* | Infection | References |
|---|---|---|---|
| ***Allium sativum* L.** | Amaryllidaceae | Common cold; COVID19; Rhinoviruses. | [48-50] |
| *Echinacea purpurea*<br>*Echinacea angustifolia* | Asteraceae | Common cold; Coronavirus 229E and SARS-CoV-2; Rhinovirus colds. | [51-61] |
| ***Eucalyptus globulus* Labill.** | Myrtaceae | Acute respiratory infection. | [62-69] |
| ***Grindelia robusta* Nutt**<br>***Grindelia squarrosa* (Pursh) Dunal**<br>***Grindelia humilis* Hook. et Arn.**<br>***Grindelia camporum* Greene** | Asteraceae | Acute respiratory infection. | [15]<br>[70-72] |

| | | | |
|---|---|---|---|
| *Glycyrrhiza glabra* **L.** *Glycyrrhiza inflata* **Bat.** *Glycyrrhiza uralensis* **Fisch** | Fabaceae | upper respiratory infections; common colds. | [73-76] |
| ***Mentha*** × ***piperita*** L. | Lamiaceae | common colds; respiratory syncytial virus (RSV). | [77-82] |
| *Origanum dictamnus* **L.** | Lamiaceae | Upper respiratory infections. | [82-84] |
| *Pelargonium sidoides* **DC** *Pelargonium reniforme* **Curt.** | Geraniaceae | Common cold; Acute respiratory tract infections. | [85-90] |
| *Pimpinella anisum* **L.** | Apiaceae | Expectorant; Common cold. | [91,92] |
| *Primula elatior* **(L.) Hill** *Primula veris* **L.** | Primulaceae | Antitussive | [93-96] |

| | | | |
|---|---|---|---|
| *Sambucus nigra* | Adoxaceae | Common cold and influenza (A and B). | [97-102] |
| ***Sideritis scardica* Griseb.**<br>***Sideritis clandestina* (Bory & Chaub.) Hayek./ *Sideritis raeseri* Boiss./ & Heldr.**<br>***Sideritis syriaca* L.** | Lamiaceae | Bronchitis; bronchial asthma;common colds. | [103-105] |
| ***Thymus vulgaris* L.,**<br>***Thymus zygis* L.** | Lamiaceae | Antitussive; common cold; Human rhinovirus. | [17][22] [106-124] |
| ***Tilia cordata* Miller**<br>***Tilia platyphyllos* Scop.**<br>***Tilia* × *vulgaris* Heyne** | Tiliaceae | Common cold. | [125] |
| ***Verbascum thapsum* L.**<br>***Verbascum densiflorum* Bertol.** | Scrophulariaceae | Common colds; coughs; Asthma; Bronchitis | [126-130] |

| | | | |
|---|---|---|---|
| ***Verbascum phlomoides* L.** | | | |
| ***Foeniculum vulgare* Miller subsp. *vulgare* var. *vulgare*** | Apiaceae | Expectorant. | [131] |
| ***Matricaria recutita* L.** | Asteraceae | Antitussive;<br>Bronchitis;<br>Fever;<br>Colds. | [76][132] |
| ***Polygonium aviculare* L.**<br>***Polypodium vulgare* L.** | Polygonaceae | Common colds. | [133] |
| ***Salix purpurea* L.**<br>***Salix daphnoides* Vill.**<br>***Salix fragilis* L.** | Salicaceae | Common colds. | [134] |
| ***Aloe arborescens*** | Asphodelaceae | | [135-137] |

| | | | |
|---|---|---|---|
| | | Upper respiratory tract infections; Human rhinovirus B (HRV14), influenza A virus (H1N1) and (H3N2), influenza B, respiratory syncytial virus (RSV), parainfluenza type 3 virus (Para 3). | |
| ***Boehmeria jamaicensis*** | Urticaceae | Common colds. | [138] |
| ***Camellia sinensis* (L.) Kuntze** ***Camellia assamica* var. *kucha*** | Theaceae | Anti-Influenza viral adsorption and suppressed replication; Cold viruses; Common cold. | [139-140] |
| ***Cistus* × *incanus*** L. | Cistaceae | Common colds; upper respiratory tract; Anti-Influenza. | [141-146] |
| ***Cinnamomum cassia*** | Lauraceae | Common cold; Chronic bronchitis; | [147] |

| | | | |
|---|---|---|---|
| | | Human respiratory syncytial virus. | |
| *Larix decidua* **Mill.** | Pinaceae | Common cold. | [148-150] |
| *Paeonia lactiflora* | Fabaceae | Rhinoviruses. | [151,152] |
| **Herbal combination** | | | |
| **Plants** | Family | Infection. | References |
| *Hedera helix/ Primula vulgaris/Thymus vulgaris* | Araliaceae/ Primulaceae/ Lamiaceae | Common cold. Acute bronchitis. | [21-23][117,118][153] |
| **Tsumura bakumondoto:** | | Common cold. | [154] |
| **Ophiopogon tuber (*Ophiopogon japonicus*)** | Asparagaceae | | |
| **Brown rice (*Oryza sativa*)** | Poaceae | | |
| **Pinellia tuber (*Pinellia ternata*)** | Araceae | | |
| | Rhamnaceae | | |
| **Jujube (*Ziziphus jujuba*)** | Fabaceae | | |
| Liquiritiae radix **(*Glycyrrhiza lepidota*)** | | | |
| | Araliaceae | | |
| **Ginseng** radix | | | |

| | | | |
|---|---|---|---|
| **(*Panax ginseng*)** | | | |
| **Gelo Myrtol®**<br>**eucalyptus**<br>**(*Eucalyptus grandis*)**<br>**sweet orange**<br>**(*Citrus sinensis (L.) Osbeck*)**<br>**myrtle**<br>**(*Myrtus communis*)**<br>**lemon**<br>**(*Citrus limon*)** | Myrtaceae<br>Rutaceae<br>Myrtaceae<br>Rutaceae | Common cold. | [155] [156] |
| Soshiho-tang:<br>**(Oriental herbal formulain East Asian countries)**<br>**Bupleuri Radix (*Bupleurum falcatum* Linne)**<br>**Pinelliae Tuber (*Pinellia ternate* Breitenbach)**<br>**Zingiberis Rhizoma Crudus (*Zingiber officinale* Roscoe)**<br>**Scutellariae Radix (*Scutellaria baicalensis* Georgi)**<br>**Ginseng Radix (Panax ginseng C.A.Meyer)**<br>**Glycyrrhizae Radix et Rhizoma (*Glycyrrhiza uralensis* Fischer)**<br>**Zizyphi Fructus** | Umbelliferae<br>Araceae<br>Zingiberaceae<br>Labiatae<br>Araliaceae<br>Leguminosae<br>Rhamnaceae | Common cold; (chills and fever) Pulmonary disease. | [157][158] |

| | | | |
|---|---|---|---|
| **(*Zizyphus jujube* Miller var. *inermis* Rehder)** | | | |
| **Kan Jang ®** **_Justicia adhatoda L._ leaf, _Echinacea purpurea_ (L.) Moench root, and _Eleutherococcus senticosus_ (Rupr. & Maxim.) Harms root.** | Acanthaceae | Respiratory tract infection. | [159] |
| **So-cheong-ryong-tang:** | Araceae | Common cold. | [160] |
| **Pinelliae tuber (tuber of _Pinellia ternata_),** | Ephedraceae | | |
| **Ephedrae herba (stem of _Ephedra sinica_),** | Schisandraceae | | |
| **Schizandrae fructus (fruit of _Schisandra chinensis_),** | Ranunculaceae | | |
| **Paeoniae radix (root of _Paeonia lactiflora_),** | Fabaceae | | |
| **Glycyrrhizae radix (root of _Glycyrrhiza uralensis_),** | Zingiberaceae | | |
| **Zingiberis Siccatum rhizoma (scalded rhizome** | Aristolochiaceae | | |

| | | | |
|---|---|---|---|
| **of *Zingiber officinale*), Asiasari radix (root of *Asiasarum heterotropoides var. mandshuricum*), and Cinnamomi cortex (bark of *Cinnamomum cassia*)** | Lauraceae | | |
| **Yeon-gyo-pae-dok-san: Forsythiae fructus (fruit of *Forsythia viridissima*), Lonicerae flos (flower of *Lonicera japonica*), Schizonepetae spica (peduncle of *Schizonepeta tenuifolia*), Ledebouriellae radix (root of *Ledebouriella divaricata*), Bupleuri radix (root of *Bupleurum falcatum*), Angenlicae Decursivae radix (root of *Angelica decursiva*), Osterici radix (root of *Ostericum koreanum*), Araliae Continentalis radix (root of *Aralia continentalis*), Aurantii fructus (fruit of *Citrus aurantum*), Platycodonis radix (root of *Planticodon grandiflorum*), Cnidii rhizoma (rhizome of *Cnidium officianale*), Poria sclerotium (sclerotia of *Poria*), Menthae herba (stem of *Mentha arvensis*), Glycyrrhizae radix (root of *Glycyrrhiza uralensis*), and Zingiberis rhizoma crudus (raw rhizome of Zingiber officinale)** | Oleaceae<br>Caprifoliaceae<br>Labiatae<br>Umbelliferae<br>Mentheae<br>Apiaceae<br>Araliaceae<br>Rutaceae<br>Apiaceae<br>Polyporaceae<br>Lamiaceae<br>Leguminosae<br>Zingiberaceae | | |
| *Radix echinaceae,*<br>*Radix baptisiae,*<br>*Herba thujae.* | Asteraceae<br>Fabaceae<br>cypress | Acute viral respiratory tract infection. | [24][161] |

| | | | |
|---|---|---|---|
| **Ma-xing-shi-gan-tang:** ***Ephedrae herba*** **(ma huang),** ***Armeniacae amarum semen*** **(ku-xing-ren),** ***Glycyrrhizae radix preparata*** **(gan-cao; licorice), and** ***Gypsum fibrosum*** **(shi-gao; calcium sulfate)** | Ephedraceae<br>Rosaceae<br>Fabaceae | Common cold;<br>Fever;<br>Influenza virus infections. | [162] |
